# Differential Pharmacological Effects on Brain Reactivity and Plasticity in Alzheimer’s Disease

**DOI:** 10.3389/fpsyt.2013.00124

**Published:** 2013-10-07

**Authors:** Anna-Katharine Brem, Natasha J. Atkinson, Erica E. Seligson, Alvaro Pascual-Leone

**Affiliations:** ^1^Berenson-Allen Center for Noninvasive Brain Stimulation, Department of Neurology, Beth Israel Deaconess Medical Center, Harvard Medical School, Boston, MA, USA; ^2^Institut Universitari de Neurorehabilitació Guttmann, Universidad Autónoma de Barcelona, Badalona, Spain

**Keywords:** Alzheimer’s disease, transcranial magnetic stimulation, brain plasticity, pharmacological therapy, acetylcholinesterase inhibitors, memantine

## Abstract

Acetylcholinesterase inhibitors (AChEIs) are the most commonly prescribed monotherapeutic medications for Alzheimer’s disease (AD). However, their underlying neurophysiological effects remain largely unknown. We investigated the effects of monotherapy (AChEI) and combination therapy (AChEI and memantine) on brain reactivity and plasticity. Patients treated with monotherapy (AChEI) (*N* = 7) were compared to patients receiving combination therapy (COM) (*N* = 9) and a group of age-matched, healthy controls (HCs) (*N* = 13). Cortical reactivity and plasticity of the motor cortex were examined using transcranial magnetic stimulation. Cognitive functions were assessed with the cognitive subscale of the Alzheimer Disease Assessment Scale-Cognitive Subscale (ADAS-Cog), activities of daily living (ADLs) with the ADCS-ADL. In addition we assessed the degree of brain atrophy by measuring brain-scalp distances in seven different brain areas. Patient groups differed in resting motor threshold and brain atrophy, with COM showing a lower motor threshold but less atrophy than AChEI. COM showed similar plasticity effects as the HC group, while plasticity was reduced in AChEI. Long-interval intracortical inhibition (LICI) was impaired in both patient groups when compared to HC. ADAS-Cog scores were positively correlated with LICI measures and with brain atrophy, specifically in the left inferior parietal cortex. AD patients treated with mono- or combination-therapy show distinct neurophysiological patterns. Further studies should investigate whether these measures might serve as biomarkers of treatment response and whether they could guide other therapeutic interventions.

## Introduction

Alzheimer’s Disease (AD) is the most common form of dementia and its treatment one of the most pressing medical issues. Current therapeutic options are limited and underlying neurophysiological mechanisms are still insufficiently understood. Available drugs mainly target cholinergic and glutamatergic pathways.

The degeneration of cholinergic neurons in the basal forebrain is thought to extensively contribute to the cognitive decline characteristic of AD (1). This led to the development of acetylcholinesterase inhibitors (AChEIs), which slow down the breakdown of acetylcholine (ACh). The resulting increase of ACh in affected forebrain regions is thought to improve cognitive functions (2, 3). AChEIs currently in use for patients with mild to moderate AD include donepezil (Aricept^®^), rivastigmine (Exelon^®^), and galantamine (Razadyne^®^), all of which show similar efficacy and side-effect profiles (4, 5).

The *N*-methyl-d-aspartate (NMDA) receptor antagonist memantine (Namenda™) is traditionally used in moderate to severe AD. At first sight it seems contradictory that blocking NMDA receptors, which play a crucial role in synaptic plasticity and therefore learning and memory, should prove efficacious in AD. Yet, bearing in mind the importance of physiological balance, both hyper- as well as hypoactivity of NMDA receptors leads to dysfunctional effects (6). As NMDA receptors are hyperactive in AD, memantine is used to re-balance glutamatergic pathways and therefore reduce neurotoxic processes.

Clinical effects of both AChEI and memantine have been extensively studied and include temporary and moderate improvements in activities of daily living (ADLs), cognitive, and behavioral functions (4, 7–9), and lead to a reduction of pathological encephalographic rhythms (10).

Several pharmacological studies have shown inconsistent results regarding the benefits of combining cholinesterase inhibitors with memantine. Tariot et al. (11) found beneficial effects of combination therapy, while consecutive studies showed no additional benefit when memantine was added to a regime of cholinesterase inhibitors (2, 12).

Transcranial magnetic stimulation (TMS) is a valuable tool to investigate the neurophysiological effects of drugs. Previous studies have examined various TMS-measures in medicated healthy subjects and in AD patients (13). In healthy subjects, memantine reduces intracortical facilitation (ICF) and enhances short-interval intracortical inhibition (SICI) in the motor cortex (MC) (14), and results in reduced MC plasticity when administered over 8 days (15). This effect is likewise observed with the NMDA antagonist dextromethorphan (16). Memantine furthermore abolishes inhibitory effects of continuous theta burst stimulation (cTBS) and facilitatory effects of intermittent theta burst stimulation (iTBS) on brain plasticity (17). AD patients treated with memantine have not yet been investigated with TMS. AChEI given to healthy subjects lead to reduced SICI and increased ICF and have no effect on motor threshold and excitability (18). Contradictory results have been found for the effect of AChEI on MC excitability in AD patients. While one study found improvements in SICI after administration of donepezil (19), another study did not find any reversal in the progressive increase of excitability during 1 year of treatment (20).

Rivastigmine furthermore leads to improvements of short latency afferent inhibition (SAI), which is associated to long-term treatment response (21). SAI is the most prominent TMS-measure impaired in AD (13, 21–23). It is mediated through GABA-ergic and cholinergic circuits (24), the latter of which plays a dominant role in AD pathology. SAI is assessed by coupling TMS and peripheral nerve stimulation of the contralateral wrist and has inhibitory interactions with long-interval intracortical inhibition (LICI) (25). To our knowledge, however, no study has investigated LICI in AD patients.

In order to understand the possible mechanisms of action of different pharmacological regimes, we evaluated MC excitability and plasticity, ADLs, cognitive functions, and brain atrophy in two groups of patients receiving either AChEI monotherapy or AChEI combined with memantine. Findings were contrasted against an age-matched healthy control (HC) group on no medications.

## Materials and Methods

### Subjects and inclusion/exclusion criteria

**Figure 1:**
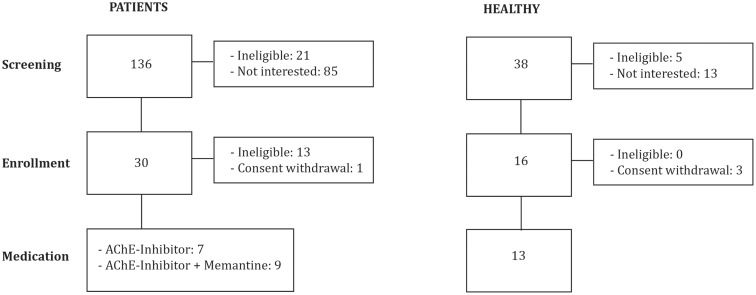
**Flow diagram of the enrollment process and final study participants analyzed**.

We investigated 16 patients diagnosed with mild to moderate AD (DSM IV, NINCDS-ADRDA): 7 were stable on AChEI (Table 1; Figure 1), and 9 were treated with combination therapy (AChEI and memantine). Patients were eligible if they had a score of 1 on the Clinical Dementia Rating scale (CDR), and attained between 18 and 24 points on the Mini-Mental State Examination (MMSE). Patients were recruited from other studies investigating AD patients (ClinicalTrials.gov NCT01504958) in comparison to older HCs. Patients were grouped according to psychopharmacological treatment. All experimenters were blinded with regards to pharmacological treatment.

**Table 1:** **Demographic, pharmacological, neuropsychological, morphometric, and neurophysiological features of study participants**.

	AChEI and memantine (*N* = 9) mean ± SD	AChEI (*N* = 7) mean ± SD	*p* Value	Healthy subjects (*N* = 13) mean ± SD	*p* Value
Age (years)	71.78 ± 3.73	68.00 ± 7.55	0.351	67.762 ± 6.05	0.278
Gender	Six female, three male	Five female, two male	1.000	Seven female, six male	0.700
Education (years)	15.33 ± 3.97	17.71 ± 3.90	0.470	15.77 ± 2.17	0.659
Duration of disease (months)	38.00 ± 34.90	16.43 ± 15.51	0.071	–	–
Duration of treatment (months)	33.00 ± 34.76	15.57 ± 16.26	0.244	–	–
Brain-scalp distance mean (mm)	16.94 ± 2.52	19.35 ± 2.93	0.114	15.78 ± 2.33	**0.014**
Brain-scalp distance left MC (mm)	15.67 ± 2.02	19.38 ± 3.78	**0.042**	14.47 ± 1.80	**0.007**
Brain-scalp distance left IPL (mm)	21.98 ± 5.35	26.39 ± 6.51	0.125	18.03 ± 2.82	**0.023**
rMT	36.83 ± 6.53	50.38 ± 8.16	**0.001**	45.26 ± 11.98	**0.012**
aMT	39.96 ± 6.07	48.43 ± 10.39	0.055	44.89 ± 8.01	0.112
MMSE	20.56 ± 2.65	23.43 ± 1.62	0.055	29.46 ± 0.88	**0.000**
ADCS-ADL	68.57 ± 5.22	72.29 ± 6.47	0.209	74.62 ± 3.59	**0.043**
GDS	2.22 ± 2.54	1.71 ± 2.06	0.918	0.69 ± 1.11	0.202
ADAS-Cog	28.21 ± 11.02	18.09 ± 6.25	0.091	4.13 ± 2.13	**0.000**
SICI	0.71 ± 0.47	0.52 ± 0.43	0.368	0.55 ± 0.69	0.360
ICF	1.27 ± 0.61	1.68 ± 1.25	0.758	1.54 ± 0.63	0.614
LICI	0.41 ± 0.52	0.98 ± 1.12	0.470	0.10 ± 0.20	**0.018**
MC reactivity (μV)	1626 ± 1418	1596 ± 1558	0.758	17.71 ± 3.90	0.907
Plasticity at T5	1.02 ± 0.30	0.78 ± 0.31	0.081	1.45 ± 0.79	**0.034**
Mean plasticity	1.06 ± 0.41	0.85 ± 0.31	0.232	1.34 ± 0.52	**0.046**

MC, motor cortex; IPL, inferior parietal lobule; rMT/aMT, resting/active motor threshold; MMSE, mini-mental state examination; ADCS-ADL, Alzheimer’s disease cooperative study-activities of daily living inventory; GDS, geriatric depression scale; ADAS-Cog, Alzheimer’s disease assessment scale-cognitive subscale; SICI, short-interval intracortical inhibition; ICF, intracortical facilitation; LICI, long-interval intracortical inhibition; *p*-values are two-tailed. Mann–Whitney *U* was used to compare patient groups, Kruskal–Wallis was used to compare all three groups. Group values are presented as mean ± standard deviation (SD). Bold font indicates significant p-values.

Alzheimer’s disease groups were compared to a group of 13 age-matched HCs with normal neurological and cognitive exams and a score of 0 on the CDR. The local Institutional Review Board approved the study protocol, and all participants or their legally authorized representatives gave written informed consent prior to study onset. The study visits took place at the Berenson-Allen Center for Non-invasive Brain Stimulation and the Harvard-Thorndike Clinical Research Center at Beth Israel Deaconess Medical Center in Boston, MA, USA. Patients with unstable or chronic medical conditions, major structural or vascular abnormalities on MRI, severe agitation, functional psychiatric disorders, a history of substance abuse or recent withdrawal, epilepsy, other progressive neurological disorders, other diseases causing memory impairment, or intake of drugs listed as a potential hazard for the application of rTMS (26) were excluded from the study.

### Assessments

Brain excitability and plasticity, brain-scalp distances, the MMSE, (ADCS-ADL), and the Geriatric Depression Scale (GDS) were assessed by trained research personnel. Certified neuropsychologists and neurologists assessed Alzheimer disease assessment scale-cognitive subscale (ADAS-Cog) and CDR.

### Mapping, brain reactivity, and plasticity measures

The Nexstim system (eXimia NBS 4) was used for neuronavigated single- and paired-pulse stimulation over the left MC. Resting motor threshold (rMT) was defined as the minimum stimulation intensity required to elicit a motor evoked potential (MEP) in the first dorsal interosseous muscle (FDI) of at least 50 μV in 5 out of 10 trials. Active motor threshold (aMT) was defined as the minimal stimulation intensity required to elicit an MEP in 5 out of 10 trials during isometric contraction of the FDI muscle. MEPs were recorded using 30 mm × 22 mm, wet gel surface electrodes (Ambu). The active electrode was placed over the muscle belly and the reference electrode over the proximal interphalangeal joint of the index finger.

Stimulation intensity for single-pulse TMS was set to 120% of rMT. For paired-pulse TMS, intensity was set to 80% rMT for the conditioning pulse and 120% rMT for the test pulse, with an inter-pulse interval of 3 ms to determine SICI and 12 ms to determine ICF. Two pulses at 120% rMT with an inter-pulse interval of 100 ms were used to determine LICI. Paired-pulse measures (each set 50 paired-pulses) were expressed as the ratio of the mean conditioned MEP amplitude to the mean unconditioned MEP amplitude.

To assess the mechanisms of cortical plasticity, neuronavigated intermittent theta burst stimulation (iTBS) was applied using the MagPro X100 (MagVenture). Baseline single-pulse stimulations (90 pulses at 120% rMT with biphasic coil) were followed by iTBS (600 pulses in 2 s trains at 50 Hz repeated every 10 s; stimulation intensity set to 80% of aMT). Sets of 30 single-pulses were delivered at 120% rMT at distinct time periods post iTBS (after 5, 10, 20, 30, 60, and 90 min).

Brain reactivity was defined as the average amplitude of 90 single-pulse MEPs at baseline; brain plasticity was expressed as the ratio of averaged MEP amplitudes obtained at each of the time points after iTBS to the mean baseline MEP amplitude. Safety guidelines were strictly followed (26).

### Brain-scalp distance measurements

As an index of brain atrophy, brain-scalp distances were measured on each individual’s brain MRI (Brainsight) in seven brain regions: left hand MC, right and left dorsolateral prefrontal cortex (DLPFC), right and left inferior parietal cortex (IPL), left superior temporal gyrus (STG), and left inferior frontal gyrus (IFG). Brain regions were determined using conventional Talairach/MNI coordinates (27–29). For each brain region, three measurements were taken in both the coronal and sagittal view, and the six measurements were averaged for each region (28).

### Data analysis

Data was analyzed using non-parametric tests (SPSS 19.0 for Mac) with the statistical significance set at *p* < 0.05. Group comparisons were calculated with the Kruskal–Wallis test, Mann–Whitney *U* tests served as *post hoc* tests. Correlations were calculated using Kendall’s tau. *p*-Values are two-tailed. As this study was an exploratory trial, a formal sample size analysis was not possible.

## Results

### Group effects

Patient groups did not differ significantly in age, gender, education years, duration of disease or pharmacologic treatment, mean brain-scalp distance, MMSE, ADCS-ADL, GDS, or ADAS-Cog scores (Table 1).

**Figure 2:**
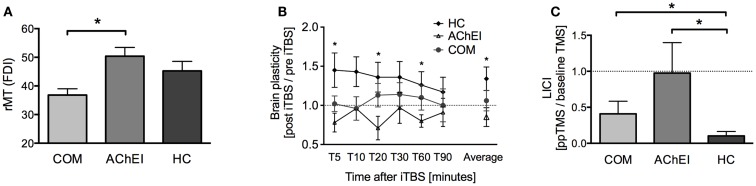
**TMS-driven measures of corticomotor reactivity and plasticity in Alzheimer’s patients treated with acetylcholinesterase inhibitors (AChEI) or a combination of AChEI and memantine (COM), and a healthy age-matched control group (HC)**. **(A)** Resting motor threshold measured from the first dorsal interosseus muscle (mean ± SE). **(B)** Brain plasticity measures (mean ± SE) over a time-period of 90 min post iTBS. **(C)** Paired-pulse TMS-measures of long-interval intracortical inhibition (LICI).

**Figure 3:**
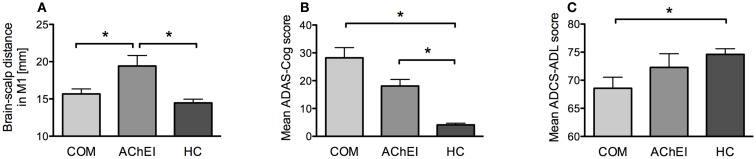
**Morphometric, neuropsychological, and behavioral measures of AChEI, COM, and HC**. **(A)** Brain atrophy as indicated by brain-scalp distances obtained from seven different brain areas (mean ± SE). **(B)** ADAS-Cog scores (mean ± SE). **(C)** ADCS-ADL scores (mean ± SE).

Acetylcholinesterase inhibitor, COM, and HC did not differ significantly regarding age, gender, education years, or GDS. However, the three groups differed significantly in rMT [*H*(2) = 8.857, *p* = 0.012], brain plasticity at T5 [*H*(2) = 6.782, *p* = 0.034], and in average degree of TBS-induced plasticity over all time points [*H*(2) = 6.146, *p* = 0.046]. Furthermore, a significant group difference was found for LICI [*H*(2) = 8.003, *p* = 0.018] (Figure 2). Brain-scalp distances were significantly different between groups when averaged over all regions [*H*(2) = 8.551, *p* = 0.014], but particularly for left IPL [*H*(2) = 7.582, *p* = 0.023], and left MC [*H*(2) = 9.852, *p* = 0.007]. In the cognitive and behavioral assessments, groups differed significantly in ADAS-Cog scores [*H*(2) = 21.713, *p* < 0.001], MMSE [*H*(2) = 22.675, *p* < 0.001], and ADCS-ADL [*H*(2) = 6.316, *p* = 0.043] (Figure 3).

### *Post hoc* group comparisons

#### Motor thresholds, brain reactivity and plasticity, brain atrophy

*Post hoc* tests revealed a significantly lower rMT in COM as compared to AChEI (*U* = 60.00, *z* = 3.02, *p* = 0.001), while rMTs of both patient groups and HC were comparable (Figure 2A). HC showed greater plasticity at T5 (*U* = 73.00, *z* = 2.18, *p* = 0.030), T20 (*U* = 74.00, *z* = 2.26, *p* = 0.024), T60 (*U* = 71.00, *z* = 2.02, *p* = 0.046), and at an average of all time points (*U* = 78.00, *z* = 2.58, *p* = 0.008) when compared with AChEI. COM and HC did not differ significantly in TBS-induced brain plasticity (Figure 2B). LICI was significantly reduced in both COM (*U* = 20.00, *z* = −2.24, *p* = 0.025) and AChEI (*U* = 12.00, *z* = −2.40, *p* = 0.015) as compared to HC (Figure 2C).

Acetylcholinesterase inhibitor showed greater atrophy as compared to HC in all three measures: averaged atrophy (*U* = 9.00, *z* = −2.89, *p* = 0.002), left IPL (*U* = 15.00, *z* = −2.42, *p* = 0.014), and MC (*U* = 7.00, *z* = −3.05, *p* = 0.001). However, AChEI also showed a significantly greater atrophy in MC as compared to COM (*U* = 51.00, *z* = 2.06, *p* = 0.042). COM did not differ from HC in atrophy (Figure 3A).

#### ADAS-Cog, MMSE, ADL, GDS

Alzheimer disease assessment scale-cognitive subscale and MMSE scores were significantly different from HC in both AChEI (ADAS-Cog: *U* = 0.00, *z* = −3.61, *p* < 0.001; MMSE: *U* = 91.00, *z* = 3.75, *p* < 0.001) and COM (ADAS-Cog: *U* = 0.00, *z* = −3.91, *p* < 0.001; MMSE: *U* = 117.00, *z* = 4.02, *p* < 0.001) (Figure 3B). COM was significantly more impaired than HC (*U* = 78.00, *z* = 2.60, *p* = 0.008) in ADCS-ADL, while HC and AChEI did not show significant differences (Figure 3C).

### Correlations (AD and HC)

**Figure 4:**
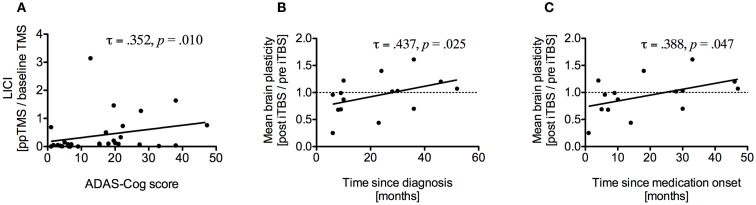
**(A)** Correlation between long-interval intracortical inhibition (LICI) and ADAS-Cog scores of all study participants. Higher ADAS-Cog scores indicate more dysfunction. **(B)** Correlation between average brain plasticity within 90 min after intermittent theta burst stimulation (iTBS) and time since diagnosis. **(C)** Correlation between average brain plasticity and time since medication onset.

Alzheimer disease assessment scale-cognitive subscale scores were significantly correlated with LICI (τ = 0.352, *p* = 0.010) (Figure 4A) and with mean atrophy over seven brain regions (τ = 0.304, *p* = 0.021), specifically with atrophy of the left IPL (τ = 0.277, *p* = 0.036). Brain-scalp distance in MC was correlated with rMT (τ = 0.287, *p* = 0.029) and aMT (τ = 0.319, *p* = 0.015) and mean brain-scalp distance was correlated with rMT (τ = 0.297, *p* = 0.024). RMT was correlated with aMT (τ = 0.688, *p* < 0.001), SICI (τ = −0.476, *p* = 0.001), and ICF (τ = −0.316, *p* = 0.022). Interestingly, disease duration was positively correlated with average plasticity measures (τ = 0.456, *p* = 0.019), specifically at T20 (τ = 0.441, *p* = 0.019). Time since medication intake was also positively correlated with average plasticity measures (τ = 0.402, *p* = 0.037). After removal of one outlier correlations remained significant for both disease duration (τ = 0.437, *p* = 0.025) (Figure 4B) and time since medication onset (τ = 0.388, *p* = 0.047) (Figure 4C).

## Discussion

We investigated the impact of pharmacological monotherapy (AChEI) and combined interventions (AChEI and memantine) on neurophysiological indices of brain reactivity and plasticity in patients with AD and age-matched HCs. Neurophysiological findings were related to cognitive function, disease duration, and measures of brain atrophy.

Patient groups showed differences in rMT and brain atrophy in MC, with COM showing lower motor thresholds but less atrophy than AChEI. Since the current induced by TMS decreases steeply with distance, a greater atrophy would be expected to result in higher MTs. Yet, most studies to date report lower MTs indicating higher motor cortical excitability in AD patients (13), but also opposite effects have been found (30). Differences in MT between COM and AChEI might therefore simply reflect differences in the degree of brain atrophy. Nonetheless, this is somewhat surprising, since greater atrophy would be expected to indicate more progressed disease stage, which might lead to the combination therapy, rather than be found in patients on monotherapy. Disease duration in COM and AChEI was however not significantly different. The addition of memantine to a regime of AChEI may therefore possibly slow down progression of atrophy; a research question that warrants further studies.

Measures of plasticity showed differences between COM and AChEI patients. COM showed similar plasticity effects as the HC group, while plasticity was reduced in AChEI. This is in line with a previous study (31) showing impaired brain plasticity in AD patients. We found no indication that the reduction in plasticity could be related to the greater atrophy in the left MC measured in AChEI. Future studies need to carefully control for atrophy and adjust stimulation intensity accordingly. However, our results suggest that atrophy does not account for the observed changes in plasticity or the implied medication effects. Disease duration, as expected, was correlated with degree of atrophy. Nonetheless, unexpectedly, we found that plasticity measures positively correlated with duration of disease and time since medication intake. Our data do not allow us to disentangle whether this unexpected finding is related to the duration of the disease or the medication treatment itself. The possibility that medication treatment might reverse an age- and disease-related trend for progressive loss of efficacy of plasticity mechanisms is intriguing. Longitudinal studies would be important to address this question.

Long-interval intracortical inhibition was the only measure that was impaired in both patient groups when compared to HC. To our knowledge this is the first study investigating LICI in AD patients. LICI and SAI have inhibitory interactions (25), which may explain our findings. SAI has been singled out as a measure reliably reduced in AD [e.g., Ref. (32)] and is a predictor of memory dysfunctions (33). Nonetheless, the mechanisms remain unclear. Evidence shows that LICI is mediated through long-lasting GABA_B_-dependent inhibitory postsynaptic potentials (IPSPs) and activation of pre-synaptic GABA_B_ receptors on inhibitory interneurons (34). Interactions between cholinergic and GABA-ergic pathways may account for our finding (35). Cognitive functions were not only positively correlated with LICI measures but also with brain atrophy, specifically in the left IPL, an area which has been previously shown to be affected early and prominently in AD patients (36). In future studies, the use of TMS combined with electroencephalography (EEG) would offer a valuable means to investigate cortical reactivity and plasticity directly in IPL and relate neurophysiological and anatomical findings more directly.

Cognitive impairments in AD have been linked to cholinergic dysfunctions (1), on the other hand, glutamate receptor hyperactivity leads to neurotoxic effects and contributes to brain atrophy (37). Studies investigating effects of memantine on brain atrophy show contradictory results. Wilkinson et al. (3) did not report any differences, while Weiner et al. (38) found that adding memantine to the AChEI regimen led to slowing of atrophic processes in the hippocampus and cognitive improvements. In our study, COM showed less brain atrophy and less plasticity aberrations than AChEI, yet they did not display better cognitive functioning or behavioral outcomes. A limitation of this study is the moderate sample size. Differences between COM and AChEI in disease duration and cognitive outcomes may therefore not be fully captured. Further prospective studies aimed at elucidating the underlying neurophysiological mechanisms of AChEI, memantine, and COM seem important, and may allow us to capitalize on pharmacologically induced physiological changes, which may be used to enhance therapeutic effects of interventions aimed at improving learning and memory, such as cognitive therapy or synergistic cognitive therapy and non-invasive brain stimulation. Future studies should moreover combine TMS with EEG in order to investigate the impact of different pharmacological regimens on brain regions outside the MC. Furthermore, brain plasticity should be measured at the onset of pharmacological treatment as well as throughout the course of the disease.

In sum, our results suggest that different pharmacological therapies can entail differential neurophysiological effects, which could affect cognitive and behavioral outcomes.

## Author Contributions

Anna-Katharine Brem, Natasha J. Atkinson, and Erica E. Seligson prepared the draft report together with Alvaro Pascual-Leone, which was critically reviewed by all authors. Anna-Katharine Brem, Natasha J. Atkinson, and Erica E. Seligson analyzed the data and contributed to data collection.

## Conflict of Interest Statement

The authors declare that the research was conducted in the absence of any commercial or financial relationships that could be construed as a potential conflict of interest. Alvaro Pascual-Leone serves on the scientific advisory boards for Nexstim, Neuronix, Starlab Neuroscience, Neuroelectrics, and NeoSync; and is listed as an inventor on several issued and pending patents on the real-time integration of transcranial magnetic stimulation (TMS) with electroencephalography (EEG) and magnetic resonance imaging (MRI).

## References

[1] Bartus RT Dean RL III Beer B Lippa AS The cholinergic hypothesis of geriatric memory dysfunction. Science (1982) 217:408–14 10.1126/science.7046051 7046051

[2] Howard R McShane R Lindesay J Ritchie C Baldwin A Barber R Donepezil and memantine for moderate-to-severe Alzheimer’s disease. N Engl J Med (2012) 366:893–903 10.1056/NEJMoa1106668 22397651

[3] Wilkinson D Schindler R Schwam E Waldemar G Jones RW Gauthier S Effectiveness of donepezil in reducing clinical worsening in patients with mild-to-moderate Alzheimer’s disease. Dement Geriatr Cogn Disord (2009) 28:244–51 10.1159/000241877 19786776 PMC3202931

[4] Birks J Cholinesterase inhibitors for Alzheimer’s disease. Cochrane Database Syst Rev (2006) (1) 10.1002/14651858.CD005593 16437532 PMC9006343

[5] Hansen RA Gartlehner G Webb AP Morgan LC Moore CG Jonas DE Efficacy and safety of donepezil, galantamine, and rivastigmine for the treatment of Alzheimer’s disease: a systematic review and meta-analysis. Clin Interv Aging (2008) 3:211–25 18686744 PMC2546466

[6] Parsons CG Stöffler A Danysz W Memantine: a NMDA receptor antagonist that improves memory by restoration of homeostasis in the glutamatergic system – too little activation is bad, too much is even worse. Neuropharmacology (2007) 53:699–723 10.1016/j.neuropharm.2007.07.013 17904591

[7] McShane R Areosa Sastre A Minakaran N Memantine for dementia. Cochrane Database Syst Rev (2006) (2) 10.1002/14651858.CD003154.pub5 16625572

[8] Winblad B Gauthier S Aström D Stender K Memantine benefits functional abilities in moderate to severe Alzheimer’s disease. J Nutr Health Aging (2010) 14:770–4 10.1007/s12603-010-0122-x 21085908 PMC12880328

[9] Yang Z Zhou X Zhang Q Effectiveness and safety of memantine treatment for Alzheimer’s disease. J Alzheimers Dis (2013) 36:445–58 10.3233/JAD-130395 23635410

[10] Babiloni C Del Percio C Bordet R Bourriez J-L Bentivoglio M Payoux P Effects of acetylcholinesterase inhibitors and memantine on resting-state electroencephalographic rhythms in Alzheimer’s disease patients. Clin Neurophysiol (2013) 124:837–50 10.1016/j.clinph.2012.09.017 23098644

[11] Tariot PN Farlow MR Grossberg GT Graham SM McDonald S Gergel I Memantine treatment in patients with moderate to severe Alzheimer disease already receiving donepezil: a randomized controlled trial. JAMA (2004) 291:317–24 10.1001/jama.291.3.317 14734594

[12] Porsteinsson AP Grossberg GT Mintzer J Olin JT Memantine MEM-MD-12 Study Group Memantine treatment in patients with mild to moderate Alzheimer’s disease already receiving a cholinesterase inhibitor: a randomized, double-blind, placebo-controlled trial. Curr Alzheimer Res (2008) 5:83–9 10.2174/156720508783884576 18288936

[13] Freitas C Mondragón-Llorca H Pascual-Leone A Noninvasive brain stimulation in Alzheimer’s disease: systematic review and perspectives for the future. Exp Gerontol (2011) 46:611–27 10.1016/j.exger.2011.04.001 21511025 PMC3589803

[14] Schwenkreis P Witscher K Janssen F Addo A Dertwinkel R Zenz M Influence of the N-methyl-D-aspartate antagonist memantine on human motor cortex excitability. Neurosci Lett (1999) 270:137–40 10.1016/S0304-3940(99)00492-9 10462113

[15] Schwenkreis P Witscher K Pleger B Malin J-P Tegenthoff M The NMDA antagonist memantine affects training induced motor cortex plasticity – a study using transcranial magnetic stimulation. BMC Neurosci (2005) 6:35 10.1186/1471-2202-6-35 15890074 PMC1134663

[16] Ziemann U Chen R Cohen LG Hallett M Dextromethorphan decreases the excitability of the human motor cortex. Neurology (1998) 51:1320–4 10.1212/WNL.51.5.1320 9818853

[17] Huang Y-Z Chen R-S Rothwell JC Wen H-Y The after-effect of human theta burst stimulation is NMDA receptor dependent. Clin Neurophysiol (2007) 118:1028–32 10.1016/j.clinph.2007.01.021 17368094

[18] Korchounov A Ilic TV Schwinge T Ziemann U Modification of motor cortical excitability by an acetylcholinesterase inhibitor. Exp Brain Res (2005) 164:399–405 10.1007/s00221-005-2326-6 15991031

[19] Liepert J Bär KJ Meske U Weiller C Motor cortex disinhibition in Alzheimer’s disease. Clin Neurophysiol (2001) 112:1436–41 10.1016/S1388-2457(01)00554-5 11459683

[20] Pennisi G Alagona G Ferri R Greco S Santonocito D Pappalardo A Motor cortex excitability in Alzheimer disease: one year follow-up study. Neurosci Lett (2002) 329:293–6 10.1016/S0304-3940(02)00701-2 12183034

[21] Di Lazzaro V Oliviero A Pilato F Saturno E Dileone M Marra C Neurophysiological predictors of long term response to AChE inhibitors in AD patients. J Neurol Neurosurg Psychiatry (2005) 76:1064–9 10.1136/jnnp.2004.051334 16024879 PMC1739760

[22] Di Lazzaro V Oliviero A Tonali PA Marra C Daniele A Profice P Noninvasive in vivo assessment of cholinergic cortical circuits in AD using transcranial magnetic stimulation. Neurology (2002) 59:392–7 10.1212/WNL.59.3.392 12177373

[23] Nardone R Golaszewski S Ladurner G Tezzon F Trinka E A review of transcranial magnetic stimulation in the in vivo functional evaluation of central cholinergic circuits in dementia. Dement Geriatr Cogn Disord (2011) 32:18–25 10.1159/000330016 21822020

[24] Di Lazzaro V Oliviero A Profice P Pennisi MA Di Giovanni S Zito G Muscarinic receptor blockade has differential effects on the excitability of intracortical circuits in the human motor cortex. Exp Brain Res (2000) 135:455–61 10.1007/s002210000543 11156309

[25] Udupa K Ni Z Gunraj C Chen R Interactions between short latency afferent inhibition and long interval intracortical inhibition. Exp Brain Res (2009) 199:177–83 10.1007/s00221-009-1997-9 19730839

[26] Rossi S Hallett M Rossini PM Pascual-Leone A Safety, ethical considerations, and application guidelines for the use of transcranial magnetic stimulation in clinical practice and research. Clin Neurophysiol (2009) 120:2008–39 10.1016/j.clinph.2009.08.016 19833552 PMC3260536

[27] Price CJ Winterburn D Giraud AL Moore CJ Noppeney U Cortical localisation of the visual and auditory word form areas: a reconsideration of the evidence. Brain Lang (2003) 86:272–86 10.1016/S0093-934X(02)00544-8 12921768

[28] Rusjan PM Barr MS Farzan F Arenovich T Maller JJ Fitzgerald PB Optimal transcranial magnetic stimulation coil placement for targeting the dorsolateral prefrontal cortex using novel magnetic resonance image-guided neuronavigation. Hum Brain Mapp (2010) 31:1643–52 10.1002/hbm.20964 20162598 PMC6871247

[29] Sajonz B Kahnt T Margulies DS Park SQ Wittmann A Stoy M Delineating self-referential processing from episodic memory retrieval: common and dissociable networks. Neuroimage (2010) 50:1606–17 10.1016/j.neuroimage.2010.01.087 20123026

[30] Perretti A Grossi D Fragassi N Lanzillo B Nolano M Pisacreta AI Evaluation of the motor cortex by magnetic stimulation in patients with Alzheimer disease. J Neurol Sci (1996) 135:31–7 10.1016/0022-510X(95)00244-V 8926493

[31] Koch G Di Lorenzo F Bonní S Ponzo V Caltagirone C Martorana A Impaired LTP- but not LTD-like cortical plasticity in Alzheimer’s disease patients. J Alzheimers Dis (2012) 31:593–9 22647254 10.3233/JAD-2012-120532

[32] Di Lazzaro V Pilato F Dileone M Saturno E Oliviero A Marra C In vivo cholinergic circuit evaluation in frontotemporal and Alzheimer dementias. Neurology (2006) 66:1111–3 10.1212/01.wnl.0000204183.26231.23 16606932

[33] Young-Bernier M Kamil Y Tremblay F Davidson PSR Associations between a neurophysiological marker of central cholinergic activity and cognitive functions in young and older adults. Behav Brain Funct (2012) 8:17 10.1186/1744-9081-8-17 22537877 PMC3379946

[34] McDonnell MN Orekhov Y Ziemann U The role of GABA(B) receptors in intracortical inhibition in the human motor cortex. Exp Brain Res (2006) 173:86–93 10.1007/s00221-006-0365-2 16489434

[35] Anderson JJ Kuo S Chase TN Engber TM GABA_A_ and GABA_B_ receptors differentially regulate striatal acetylcholine release in vivo. Neurosci Lett (1993) 160:126–30 10.1016/0304-3940(93)90395-2 8247341

[36] Im K Lee J-M Seo SW Yoon U Kim ST Kim Y-H Variations in cortical thickness with dementia severity in Alzheimer’s disease. Neurosci Lett (2008) 436:227–31 10.1016/j.neulet.2008.03.032 18400396

[37] Meier-Ruge WA Bertoni-Freddari C Pathogenesis of decreased glucose turnover and oxidative phosphorylation in ischemic and trauma-induced dementia of the Alzheimer type. Ann N Y Acad Sci (1997) 826:229–41 10.1111/j.1749-6632.1997.tb48474.x 9329694

[38] Weiner MW Sadowsky C Saxton J Hofbauer RK Graham SM Yu SY Magnetic resonance imaging and neuropsychological results from a trial of memantine in Alzheimer’s disease. *Alzheimers Dement* (2011) 7:425–35 10.1016/j.jalz.2010.09.003 21646051

